# The mononuclear phagocyte system obscures the accurate diagnosis of infected joint replacements

**DOI:** 10.1186/s12967-024-05866-5

**Published:** 2024-11-19

**Authors:** Robert Manasherob, Shay I. Warren, Prerna Arora, Lyong Heo, Naomi L. Haddock, Ievgen Koliesnik, Diasuke Furukawa, Z. Ngalo Otieno-Ayayo, William J. Maloney, David W. Lowenberg, Stuart B. Goodman, Derek F. Amanatullah

**Affiliations:** 1 Department of Orthopaedic Surgery, Stanford University, 450 Broadway Street, Redwood City, CA 94025 USA; 2 Department of Orthopaedic Surgery, Stanford School of Medicine, Biomedical Innovations Building, 240 Pasteur Drive, Palo Alto, CA 94304 USA; 3 Genetics and Bioinformatics Service Center (GBSC), Stanford University, 3165 Porter Drive, Palo Alto, CA 94304 USA; 4 School of Medicine, University of California, San Francisco, 513 Parnassus Avenue, San Francisco, CA 94143 USA; 5 Division of Infectious Diseases and Geographic Medicine, Department of Medicine, Stanford University, 211 Quarry Road, Palo Alto, CA 94305 USA; 6 School of Science, Agriculture, and Environmental Studies, Rongo University, P.O. Box 103, Rongo, 40404 Kenya

## Abstract

**Introduction:**

Diagnosing infected joint replacements relies heavily on assessing the neutrophil response to bacteria. Bacteria form biofilms on joint replacements. Biofilms are sessile bacterial communities encased in a protective extracellular matrix, making them notoriously difficult to culture, remarkably tolerant to antibiotics, and able to evade phagocytosis. Phagocytized bacteria dramatically alter cytokine production and compromise macrophage antigen presentation. We hypothesize that a subset of joint replacements have a dormant infection that suppresses the neutrophil response to bacteria but can be distinguished from uninfected joint replacements by the response of the mononuclear phagocyte system (MPS) within periarticular tissue, synovial fluid, and circulating plasma.

**Methods:**

Single cell RNASeq transcriptomic and OLink proteomic profiling was performed on matched whole blood, synovial fluid, and periarticular tissue samples collected from 4 joint replacements with an active infection and 3 joint replacements without infection as well as 6 joint replacements with a prior infection deemed “infection-free” by the 2018 Musculoskeletal Infection Society criteria (follow-up of 26 ± 3 months).

**Results:**

The MPS and neutrophil responses differ by infected state; the cellular distribution of the MPS response in the subset of joints with dormant infections resembled actively infected joints (p = 0.843, Chi-square test) but was significantly different from uninfected joints (p < 0.001, Chi-square test) despite the absence of systemic acute phase reactants and recruitment of neutrophils (p < 0.001, t-test). When compared to no infection, the cellular composition of dormant infection was distinct. There was reduction in classically activated M1 macrophages (p < 0.001, Fischer's test) and alternatively activated M2 macrophages coupled with an increase in classical monocytes (p < 0.001, Fischer’s test), myeloid dendritic cells (p < 0.001, Fischer’s test), regulatory T-cells (p < 0.001, Fischer’s test), natural killer cells (p = 0.009, Fischer’s test), and plasmacytoid dendritic cells (p = 0.005, Fischer’s test). Hierarchical cluster analysis and single-cell gene expression revealed that classically M1 and alternatively M2 activated macrophages as well as myeloid dendritic cells can independently distinguish the dormant and uninfected patient populations suggesting that a process that modulates neutrophil recruitment (C1QA, C1QB, LY86, SELL, CXCL5, CCL20, CD14, ITGAM), macrophage polarization (FOSB, JUN), immune checkpoint regulation (IFITM2, IFITM3, CST7, THBS1), and T-cell response (VISIG4, CD28, FYN, LAT2, FCGR3A, CD52) was occurring during dormant infection. Gene set variation analysis suggested that activation of the TNF (FDR < 0.01) and IL17 (FDR < 0.01) pathways may distinguish dormant infections from the active and uninfected populations, while an inactivation of neutrophil extracellular traps (NETs) may be involved in the lack of a clinical response to a dormant infection using established diagnostic criteria. Synovial inflammatory proteomics show an increase in synovial CXCL5 associated with dormant infection (p = 0.011, t-test), suggesting the establishment of a chronic inflammatory state by the MPS during a dormant infection involved in neutrophil inhibition. Plasma inflammatory proteomics also support a chronic inflammatory state (EGF, GZMN, FGF2, PTN, MMP12) during dormant infection that involves a reduction in neutrophil recruitment (CXCL5, p = 0.006, t-test), antigen presentation (LAMP3, p = 0.047, t-test), and T-cell function (CD28, p = 0.045, t-test; CD70, p = 0.002, t-test) that are also seen during the development of bacterial tolerance.

**Discussion:**

All current diagnostic criteria assume each patient can mount the same neutrophil response to an implant-associated infection. However, the state of the MPS is of critical importance to accurate diagnosis of an implant-associated infection. A reduction in neutrophil recruitment and function mediated by the MPS may allow joint replacements with a dormant infection to be mischaracterized as uninfected, thus limiting the prognostic capabilities of all current diagnostic tests.

**Supplementary Information:**

The online version contains supplementary material available at 10.1186/s12967-024-05866-5.

## Introduction

**Fig. 1:**
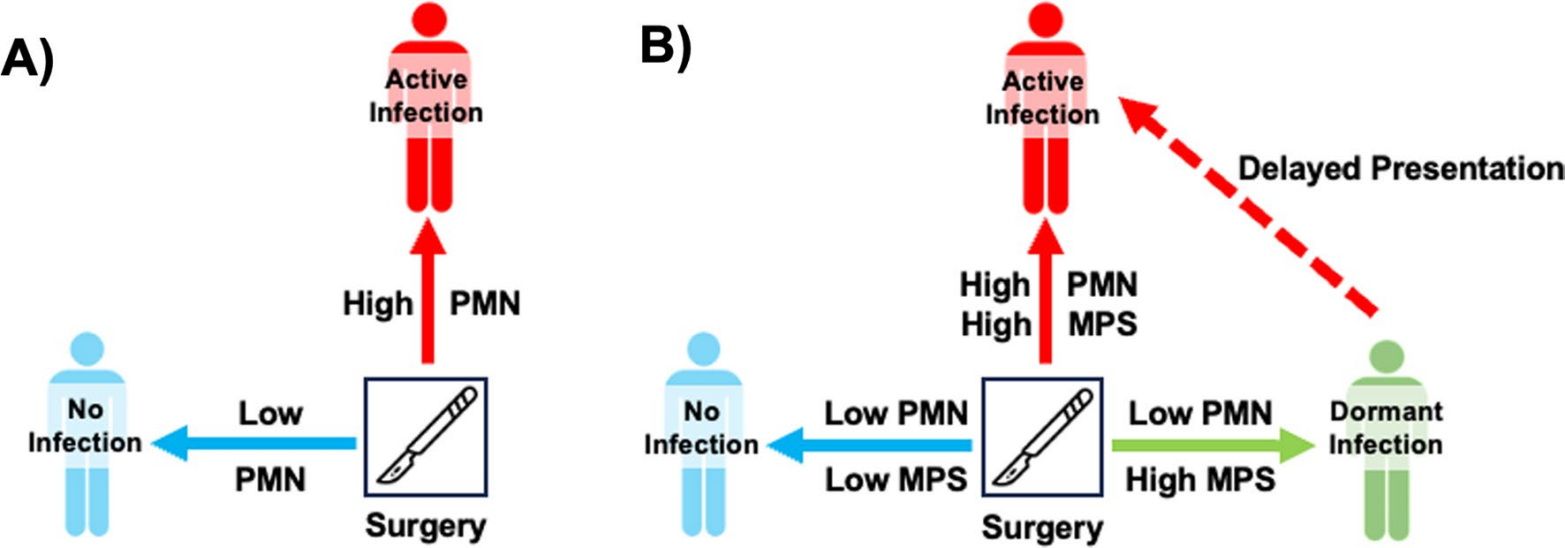
**A** The local and systemic polymorphonuclear neutrophil (PMN) response is used to diagnose active infection and drive surgical management. **B** The PMN response is part of a more complex innate immune response to bacteria regulated by the mononuclear phagocyte system (MPS). We hypothesize that a subset of joint replacements harbors a dormant infection that can be distinguished from uninfected joint replacements by the MPS response to bacteria

The accurate diagnosis of an infected joint replacement relies primarily on the systemic and local neutrophil response to bacteria. Current diagnostic tests for a joint replacement with an active infection include standard microbiological synovial fluid and tissue cultures, systemic acute phase reactants present during the neutrophil response [e.g., erythrocyte sedimentation rate (ESR), C-reactive protein (CRP), D-dimer], and the synovial neutrophil response [e.g., synovial white blood cell count (sWBC), percentage of synovial polymorphonuclear neutrophils (PMN%), double positive leukocyte esterase strips, histologic presence of PMNs within the synovium, synovial alpha-defensin (SaD) expression normalized to synovial CRP] [1, 2]. The systemic and local neutrophil response to bacteria is highly specific for diagnosing an active infection (Fig. 1A) [3–5] but are highly redundant and may not necessarily add independent diagnostic value, creating a false elevation of sensitivity, specificity, and predictive value [6]. After surgery, it can be difficult to distinguish the neutrophil response to bacteria from the neutrophil response stimulated by the acute insult of surgery itself, since 40% of uninfected joint replacements have a persistent neutrophil response for more than 6 weeks [7–9].

The presentation of an infected joint replacement is highly variable, dictated by timing after surgery, infecting organism, host immune status, and mechanism of infection. Traditionally, early infection is defined within 1 month (Tsukayama definition) [10] or within 90 days (Center for Disease Control definition) after surgery [11]. Early infection presents with pain, swelling, surrounding erythema, tenderness, fevers, and wound drainage, and there is often little debate about the presence of an early infection. However, late infections result from three pathological mechanisms. First, direct surgical inoculation with a low virulence organism that emerges in a delayed fashion. Second, indirect inoculation due to acute hematogenous spread. Third, direct surgical inoculation with bacteria that avoid eradication and emerge in a delayed fashion (a.k.a., dormant infection) after new trauma/surgery, a change in the global immune status of the host, or quorum triggering (Fig. 1B). An early infection within 4 weeks of surgery and the acute presentation of a late hematogenous infection with less than 4 weeks of symptoms are often managed with debridement and implant retention (DAIR) with reported success rates ranging widely from approximately 30–80% [11, 12]. It is likely that the high failure rate observed in DAIR is due in part to the misdiagnosis of a now emerging dormant infection as a new acute hematogenous infection. Moreover, the relatively high failure rate of DAIR may be caused by biofilm-mediated bacterial tolerance, which is present even after implant removal. An increased focus on the immunology of dormant infection may improve clinical decision-making and provide potential therapeutic targets to improve these outcomes.

The neutrophil response used by current diagnostic tests is part of a larger and more complex innate immune response to bacteria regulated by the mononuclear phagocyte system (MPS). The MPS consists of local macrophages, resident dendritic cells, and circulating monocytes that regulate the innate immune system’s response to bacteria. In response to an infected joint replacement, the MPS initiates a pro-inflammatory immune response by secreting cytokines [e.g., interleukin (IL) 1, IL6, IL8, macrophage inflammatory proteins] that recruit neutrophils and activate macrophages to eradicate bacteria [13]. The MPS concurrently initiates an anti-inflammatory immune response by secreting a different set of cytokines (e.g., IL4, IL13, IL10, IL35, transforming growth factor beta (b), indoleamine 2,3-dioxygenase) [14–17] and major histocompatibility class II costimulatory immune checkpoint molecules [e.g., programmed death ligand (PD-L) 1] that drive myeloid-derived suppressor cell formation as well as T-cell function via anergy, depletion, or conversion of naïve T-cells to regulatory T-cells [18]. The anti-inflammatory response of the MPS is responsible for the resolution of the initial pro-inflammatory response as well as bacterial tolerance (i.e., not eradicating bacteria). Bacterial tolerance is critical, since it prevents the development of autoimmunity to commensal organisms that provide essential nutrients and probiotic metabolites [19–22]. Because current diagnostic tests for an infected joint replacement are focused solely on the neutrophil response to bacteria present during bacterial eradication, the tests can only accurately identify the incidence of active infection over time (Fig. 1A). All current diagnostic tests neglect the possibility of a novel class of joint replacements with a dormant infection that are essential for the development of novel screening tests and disease prevention (Fig. 1B) [23, 24].

Bacteria form biofilms on joint replacements. Biofilms are bacterial communities encased in a protective extracellular matrix making them notoriously difficult to culture, remarkably tolerant to antibiotics, and able to evade phagocytosis [25–28]. Biofilm-resident bacteria affect the MPS by dramatically altering local cytokine production and compromising antigen presentation [29, 30]. When engulfed, biofilm-resident bacteria stimulate the production of interleukin-10 (IL10) [31–34], which polarizes macrophages from the classical activation state (M1) to an alternative activation state (M2) [28, 29, 34, 35]. Biofilm-resident bacteria also upregulate monocyte expression of PD-L1 [8, 36]. Immune checkpoint molecules, like PD-L1 and PD-L2, inhibit signaling pathways involved in antigen clearance [36–42]. Immune checkpoint molecule expression is a risk factor for the development of an active infection. In our published pilot study of 31 patients, PD-L1 expression in the macrophage infiltrate of joint replacements was associated with an eightfold increase in the odds of a prolonged neutrophil response (odds ratio: 8.3, 95% confidence interval: 0.6–110, p = 0.043) and a 22-fold increase in the odds of developing a new infection (odds ratio: 21.9, 95% confidence interval: 0.9–524, p = 0.038) [8]. Thus, it is highly plausible that biofilm-resident bacteria may establish a dormant infection by influencing the local immune response, mediated by the MPS that suppresses neutrophil recruitment. Thus, the neutrophil response to biofilm-resident bacteria may be a poor predictor of future active infections that emerge over time [43]. We hypothesize that a subset of joint replacements harbor a dormant infection that suppresses the neutrophil response to bacteria but can be distinguished from uninfected joint replacements by the response of the MPS within periarticular tissue, synovial fluid, and circulating plasma (Fig. 1B).

Numerous factors unique to each patient increase the risk of developing an infected joint replacement, including obesity, diabetes mellitus, rheumatoid arthritis, immunosuppressive medications, malignancy, smoking, injection drug use, prior bacteremia, prior septic arthritis in the operative joint, and prior arthroplasty or arthroscopy on the operative joint [44–53]. Perioperative risk factors also include active infection at distant sites, prolonged duration of the surgical procedure, allogeneic blood transfusion, and wound dehiscence [11]. All of these risk factors can be directly or indirectly tied to host immunity. Yet, the state of a patient’s MPS prior to a joint replacement remains uncharacterized in each case, rendering prognostication elusive despite ceaseless evaluation of associative clinical parameters. This highlights the fact that all current diagnostic criteria erroneously assume each patient can mount the same neutrophil response to an infected joint replacement and neglect the observation that the neutrophil response is regulated by the MPS [1]. In fact, four scenarios suggesting that patients may have dormant infections without a robust neutrophil response. First, the clearest sign of a joint replacement with an active infection is the presence of a sinus tract (e.g., a joint replacement that has chronically draining frank purulent fluid from an open wound) [1]. When a sinus tract is present, there may not be a neutrophil response to bacteria, and the infecting bacteria may be difficult to isolate using a synovial fluid culture [3]. Second, ≥ 30% of patients with a neutrophil response to bacteria never have an identified synovial fluid or tissue culture (i.e., culture-negative infections) [54]. Third, immunocompromised patients with inflammatory arthritis [55], cancer [56–58], or solid organ transplantation [59, 60] may not have a neutrophil response even when bacteria are identified by synovial fluid or tissue culture [61]. Fourth, antibiotics can be used to control the neutrophil response and even suppress synovial fluid and tissue culture results, but the use of antibiotics does not remove bacteria from a joint replacement, especially when biofilm is present [62, 63]. However, when antibiotics are stopped, the bacteria re-emerge, as does the neutrophil response [30, 54, 64–66].

## Methods

### Patient Samples

After Institutional review board approval at Stanford University (Biosaftey #4328, Clinical Safety #54462 and #72855), we collected 5 cc of whole blood in a purple top tube, aspirated synovial fluid in a purple top tube, and 5 cc of periarticular tissue from the superior capsule (total hip arthroplasty) or suprapatellar pouch (total knee arthroplasty) from 48 patients. From the attempted cohort, 20 complete matched samples were obtained (42%). Of the matched cohort, 13 samples (65%) had sufficiently high quality RNA and protein isolation for final analysis: 4 joint replacements with a *Staphylococcus aureus*, *lugdunesis*, or *epidermidis* infection (follow-up: 26 ± 5 months after resection and spacer placement), 3 joint replacements without an infection (follow-up: 27 ± 1 month after revision surgery, p = 0.621, t-test v. infections), and 6 joint replacements with a prior *S. aureus*, *S. lugdunesis*, S. *epidermidis*, or *Salmonella enterica* infection deemed “infection-free” by the 2018 Musculoskeletal Infection Society (MSIS) criteria for 14 ± 12 week prior to reimplantation and after completion of antibiotics (follow-up of 25 ± 2 months after reimplantation, p = 0.597, t-test v. infections) [1]. Comparisons of categorical variables between these groups were made with an X^2^ test, while comparisons of continuous variables were made using a Welch two-tailed unpaired t-test.

### The definition of dormant infection

We intentionally utilized an immunologic definition of a dormant infection based on a persistent MPS response after a prior bacterial infection. First, biofilm and bacterial persistence often obscure bacterial culture results making them a highly unreliable diagnostic tool [30, 67, 68]. Second, advanced sequencing techniques are subject to surgical sampling bias and the detection of contaminating bacterial DNA [69, 70]. Finally, our goal was not to define a new test to replace synovial or tissue culture but to define the local and systemic MPS response and influence the immunologic definition of infection itself. This is particularly important, because ultimately the 2018 MSIS criteria are immunologic criteria that represent the local and systemic neutrophil response only [1]. Hence, a dormant infection was defined as a joint replacement with a prior infection diagnosed by the 2018 MSIS criteria, that underwent implant resection and completion of 6 weeks of microbe-specific intravenous antibiotic therapy, that was subsequently reimplanted after being deemed infection free using the same 2018 MSIS criteria at the end of a > 6 week antibiotic holiday, and at reimplantation had a persistent local or systemic MPS response that mimics the MPS response present at the time of an active infection.

### Pseudobulk and single cell RNASeq

Raw sequence files from two different batches were processed using SevenBridges genomics workflow. The raw counts were then loaded into R using the Seurat v4.9.9 package for further data processing [71]. Cells were retained if they met the following criteria: fewer than 1 × 10^5^ unique molecular identifiers and more than three raw expression counts on CD45. No mitochondrial chromosomes were presented in the expression matrix. The Seurat objects from each batch were merged. After that, SCTransform version 2 (v0.4.0) with glmGamPoi method was performed [72, 73]. The unique molecular identifiers and gene numbers served as regression variables in SCTransform, an R package for modeling single-cell UMI expression data using regularized negative binomial regression. Next, we performed principal component analysis (PCA), and the resulting principal component (PCs) were corrected and harmonized using the Harmony algorithm to integrate datasets from different batches [74]. We then performed uniform manifold approximation and projection using the top 30 corrected PCs from Harmony and applied the Louvain algorithm to cluster the cells. Before implementing automatic cell type annotation with SingleR v2.0.0 [75], we initially extracted macrophage subtype data and labels from Blueprint/ENCODE data [75–77]. Subsequently, we created the reference set by integrating these data with the Monaco immune data [75]. We used the FindAllMarkers function with 10% of a minimum percentage in either of the two groups of cells and a minimum of 0.25 of log_2_ fold changes to find the marker genes for each cell type. Pairwise comparisons on the relative abundance in each cell type were done using the Wilcoxon rank sum test embedded in the ggpurb v0.6.0 R package. Raw read counts in the single-cell dataset were aggregated into a pseudobulk dataset by summing the counts across all cell types.

### Correlation of sample counts to cellular group

The variance in raw expression counts was stabilized using Variance-Stabilizing Transformation (VST) in the DESeq2 R package. Agglomerative hierarchical clustering of the dataset was computed using the Agnes function with Ward’s method in the cluster R package. The dataset was then transformed into lower-dimensional data using the first two principal components, PC1 and PC2.

### Pseudobulk differential expressed (DE) analysis

We first performed three DE analyses to identify DE genes in the dormant infection group compared to the infected or uninfected samples. Subsequently, we compared the dormant group against the combined infected and uninfected groups to pinpoint unique DE genes during dormant infection. For the DE analyses above, genes with a log_2_ fold change of one or more and an adjusted p-value under 0.05 (values generated by the negative binomial generalized models and Wald test employed by DESeq2 v1.42.1) were considered significant. These significant genes were then used to perform gene set enrichment analysis (GSEA) of gene ontology (GO) terms and Kyoto Encyclopedia of Genes and Genomes (KEGG) pathways.

### Gene set variation analysis (GSVA)

We utilized non-normalized expression data (integer counts) to calculate GSVA scores for pathways derived from GO terms and KEGG using the GSVA method in GSVA v1.50.5 [78]. The parameters for GSVA were set with a minimum size of 5, a maximum size of 500, and kernel density estimation set to Poisson. DE analyses were performed using the limma package for two comparisons: infected vs. dormant infection and uninfected vs. dormant infection [79]. Only pathways with a false discovery rate (FDR) < 0.01 were considered statistically significant.

### Assessment of polarization

To quantify the score of the signature gene set of M1/M2 polarization, we adopted the signature gene sets described by Sun et al. [80] and utilized the GSVA parameters detailed previously in our methods. We then performed pairwise comparisons of the three groups. For these comparisons, we utilized t-test function and adjusted p-values using adjust_pvalue function from the rstatix v0.7.2, applying the Benjamin-Hochberg correction method for controlling the FDR. Significance was determined at a strict threshold for FDR < 0.01 allowing us to identify statistically significant differences in polarization across the studied cellular groups.

### Inflammatory synovial fluid and circulating plasma proteomics

The concentration of 96 plasma proteomic analytes was determined using a highly multiplexed platform (Olink Proteomics, Immuno-Oncology Panel from intra-operative synovial fluid and pre-operative plasma samples matched to the tissue samples from each patient via proximity extension [81]. Pairwise comparisons on each proteomic analyte with respect to normalized protein expression (NPX, log_2_) were done using a Welch two-sided t-test embedded in the Olink Statistical Analysis Application. Only proteomic analytes with an adjusted p-value < 0.05 were considered statistically significant.

## Results

### The MPS and neutrophil responses differ by infected state

**Fig. 2:**
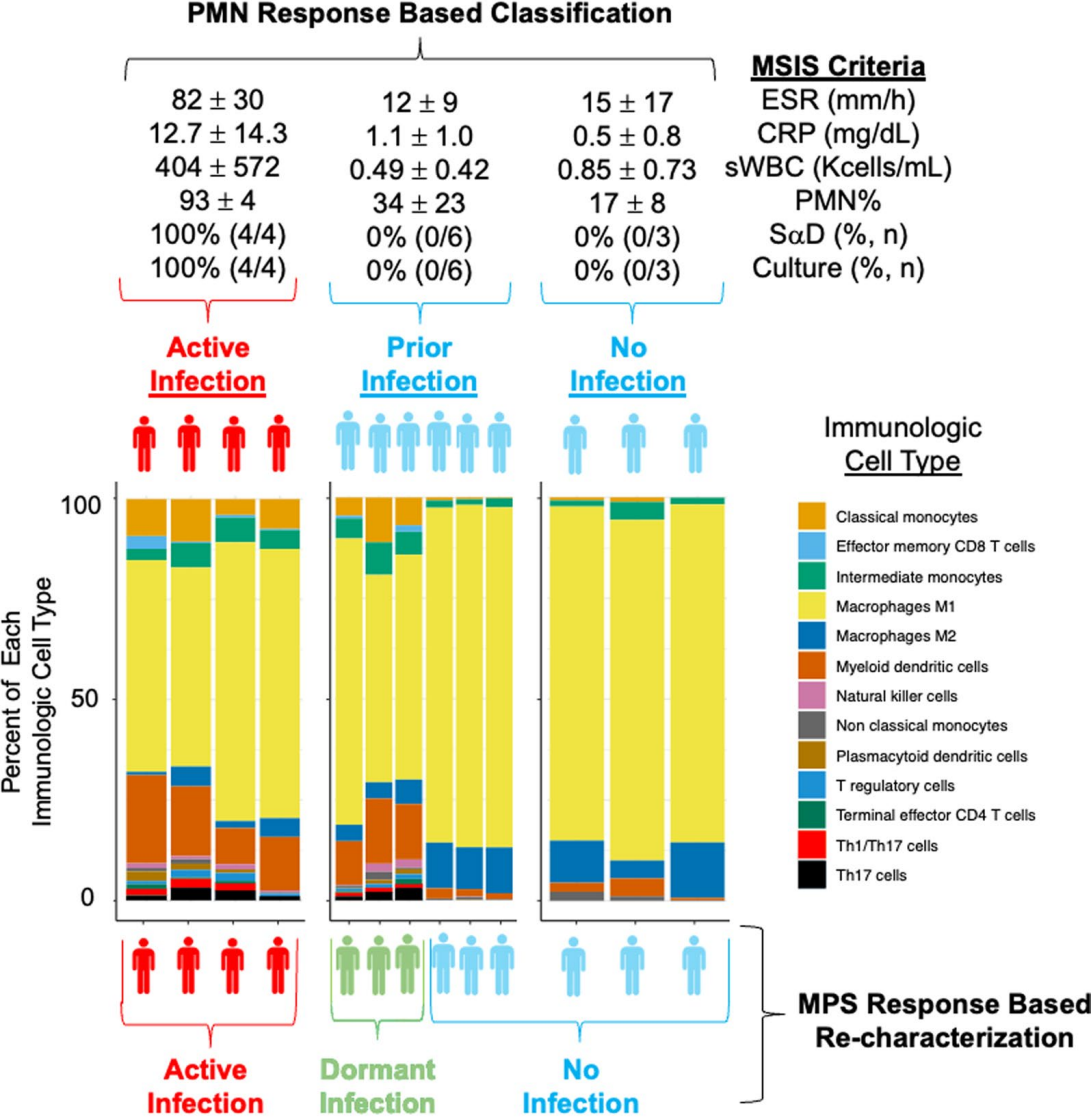
The Musculoskeletal Infection Society (MSIS) criteria were used to subdivide clinical tissue samples from patients with (red) and without (blue) an active infection (top panel). The uninfected samples included cases with and without a previous infection and are both marked in blue. Single-cell transcriptomic expression from synovial tissue was used to re-characterize each clinical tissue sample (bottom panel). Samples previously deemed “infection-free” after a prior infection using the polymorphonuclear neutrophil (PMN) response, were able to be subdivided into two subpopulations based on their immunologic phenotype. One group was statistically similar to the uninfected cases (p = 0.902, Chi-squared) and remains marked in blue, while another group, dormant infections, was statistically similar to an active infection (green, p = 0.843, Chi-squared) and is now marked in green

Our analysis compared the MPS response in local tissue samples from joint replacements with different infection statuses: active infections, previously infected joints deemed “infection-free” prior to re-implantation surgery, and uninfected controls. The cellular distribution of the MPS response in the subset of joints with dormant infections resembled that of actively infected joints (p = 0.843, Chi-square test) but was significantly different from uninfected joints (p < 0.001, Chi-square test) (Fig. 2—bottom panel). This was observed despite the absence of systemic acute phase reactants such as ESR and CRP, which showed significant differences (both p < 0.001), and the lack of synovial neutrophil recruitment as indicated by sWBC, PMN%, SαD, and culture results (all p < 0.001, t-tests) (Fig. 2—top panel). Three prior infections (3/6, 50%) seem to have an MPS response similar to an active infection, while the other three (3/6, 50%) appear have an MPS response similar to the uninfected controls. Only one patient in the dormant infection group as identified by the MPS (1/3, 33% v. dormant and 1/6, 17% v. prior infection) developed a subsequent *S. aureus* infection after reimplantation and was placed on lifelong antibiotic suppression.

### The cellular composition of dormant infection

In dormant infections, the count of classically activated M1 macrophages was significantly reduced to 168.0 ± 134.5 cells (59%), compared to 723.2 ± 895.6 cells (84%) in uninfected joints (p < 0.001, Fischer’s test) (Fig. 2—bottom panel). Alternatively activated M2 macrophages were also fewer: 12.3 ± 7.3 cells (5%) versus 105.7 ± 147.4 cells (10%) in uninfected controls (p = 0.023, Fischer’s test) (Fig. 2—bottom panel). Conversely, there was an increase in classical monocytes (16.0 ± 4.6 cells [8%] vs. 2.2 ± 1.9 cells [1%], p < 0.001, Fischer’s test), myeloid dendritic cells (32.7 ± 16.0 cells [14%] vs. 10.2 ± 7.8 cells [2%], p < 0.001, Fischer’s test), regulatory T-cells (2.3 ± 1.2 cells [1%] vs. 0.0 ± 0.0 cells [0%], p < 0.001, Fischer’s test), natural killer cells (2.7 ± 2.1 cells [1%] v. 0.3 ± 0.8 cells [0%], p = 0.009, Fischer’s test), and plasmacytoid dendritic cells (1.7 ± 1.2 cells [1%] v. 0.2 ± 0.4 cells [0%], p = 0.005, Fischer’s test) all showed a significant increase (Fig. 2—bottom panel).

### Cell type specific differential gene expression during dormant infection

**Fig. 3:**
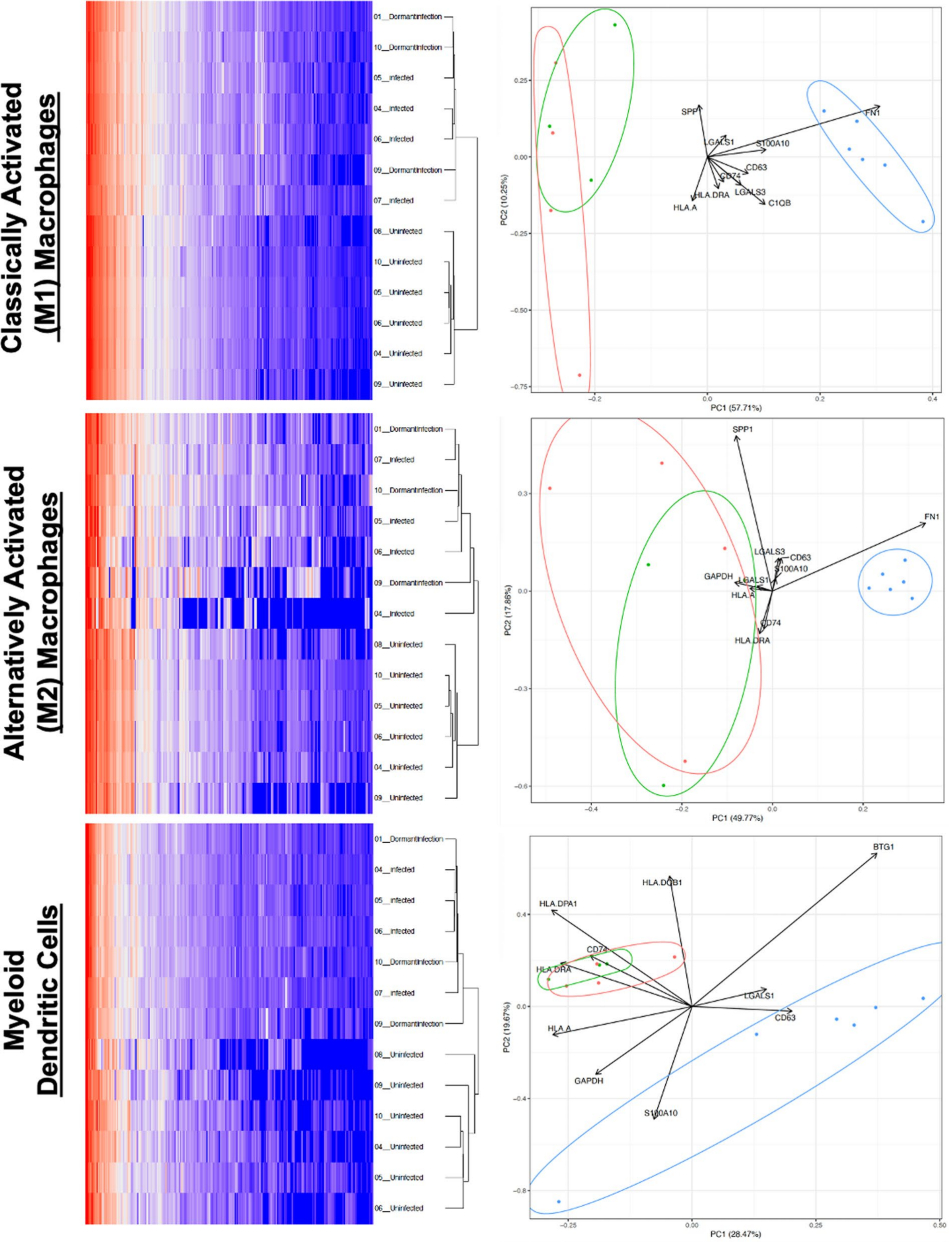
Hierarchical clustering of the single-cell gene expression from classically activated M1 and alternatively M2 activated macrophages as well as myeloid dendritic cells each independently distinguish the uninfected tissue samples (blue circle) from the infected (red circle) and dormant infection (green) samples for which the 95% confidence intervals tend to overlap demonstrating their similarity with respect to principal components (PC) 1 and 2. Loadings plot is overlayed to visualize features defining PC1 and PC2 and directionality relative to the localization of uninfected, infected, and dormant infection groups along these components

Hierarchical cluster analysis revealed that the single-cell gene expression of classically M1 and alternatively M2 activated macrophages as well as myeloid dendritic cells can independently distinguish the dormant and uninfected patient populations with myeloid dendritic cells outperforming macrophages (Fig. 3). While FN1 expression in M1 and M2 macrophages appeared to be a hallmark of uninfected tissue and expression of HLAs in both macrophage subtypes and myeloid dendritic cells appeared to be a hallmark of infection, despite none of the MPS lineages distinguishing dormant from active infection (Fig. 3).

**Fig. 4:**
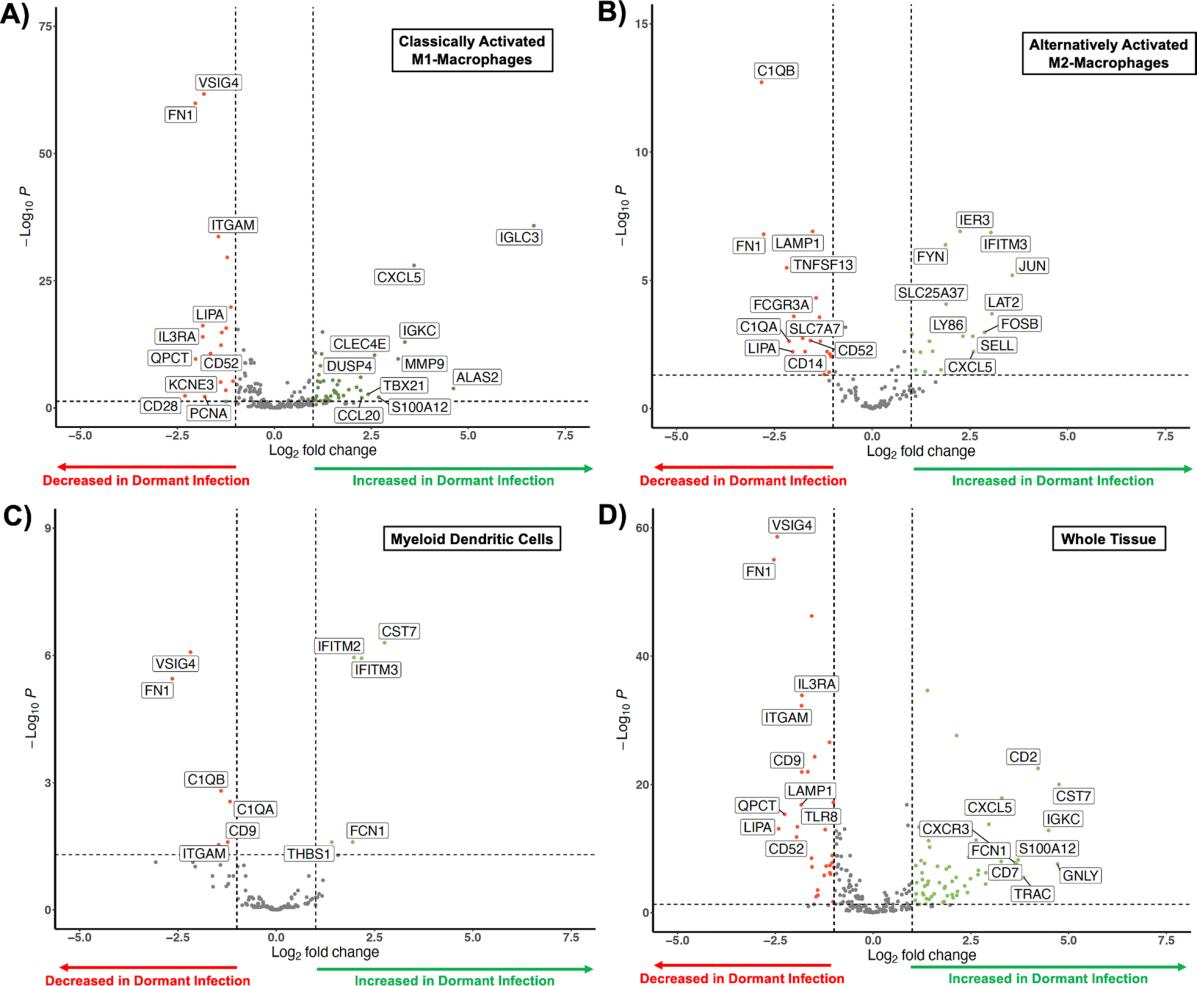
Volcano plot highlighting the significant transcriptomic changes (red dot with the top 20 transcripts labeled and the remainder of the significant transcripts are listed in Supplemental Tables 1–3; the green and grey dots represent transcripts that did not reach significance) in **A** classically activate M1 macrophages, **B** alternatively M2 activated macrophages, **C** myeloid dendritic cells, and **D** in the tissue via pseudobulk analysis. Arrows indicate genes that exceeded the presented axes and are listed in Supplemental Tables 1 and 2

The single-cell transcriptomic analysis showed significant alterations in gene expression patterns between dormant infection and uninfected controls. Classically activated M1 macrophages (Fig. 4A, Supplemental Table 1) and alternatively activated M2 macrophages (Fig. 4B, Supplemental Table 2) in dormant infections exhibited significant changes in genes related to macrophage polarization (FOSB, JUN), neutrophil recruitment (C1QA, C1QB, LY86, SELL, CXCL5, CCL20, CD14), immune checkpoint regulation (IFITM3, CST7), and T-cell response (VISIG4, CD28, FYN, LAT2, FCGR3A, CD52). Myeloid dendritic cells in dormant infections also showed notable differences in mRNA expression, affecting neutrophil recruitment (C1QA, C1QB, ITGAM), immune checkpoint regulation (IFITM2, IFITM3, CST7, THBS1), and T-cell response (VISIG4, V-set immunoglobulin domain containing 4) (Fig. 4C).

In addition, the single-cell transcriptomic analysis revealed a few alterations in gene expression patterns between dormant and active infection all involving the T-cell response. Th1/17T-cells exhibited significantly higher expression of thymidylate synthase (TYMS, 9.97 log_2_FC, p < 0.001), plasmid dendritic cells exhibited significantly higher expression of T-cell leukemia/lymphoma protein 1A (TCL1A, 8.46 log_2_FC, p < 0.001), and non-classical monocytes exhibited significantly higher expression of VSIG4 (8.20 log_2_FC, p < 0.001) in dormant infections (data not shown).

### Cellular polarization

**Fig. 5:**
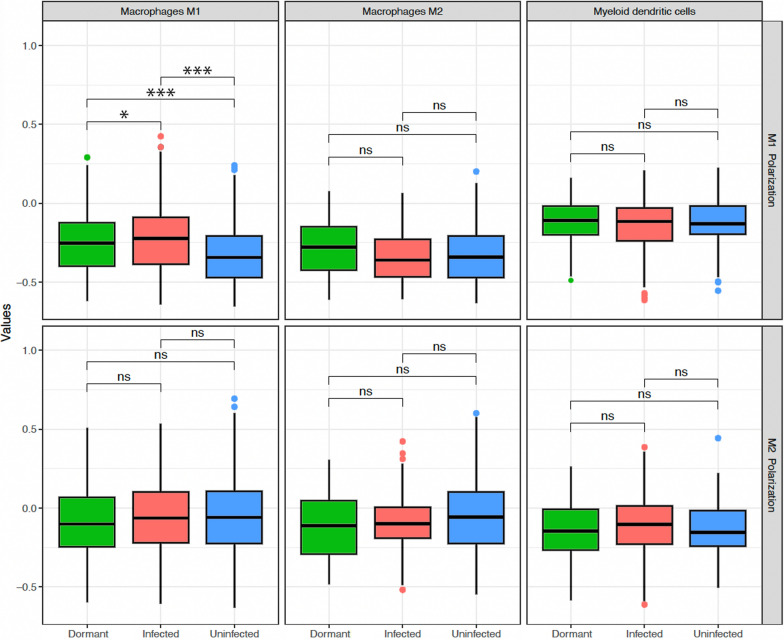
M1 (top panel) and M2 (bottom panel) polarization of M1 macrophages, M2 macrophages, and myeloid dendritic cells displayed as median and interquartile range in dormant infection (green), active infection (red), and uninfected samples (blue). The symbols in the plots represent statistical significance as follows: non-significant (ns), FDR < 0.001 (*), FDR < 0.0001 (**), and FDR < 0.0001 (***)

Within the M1 macrophage population, the magnitude of M1 polarization was higher in both dormant and active infection when compared to uninfected samples (FDR < 0.001) (Fig. 5—top panel). However, dormant infection M1 macrophages did not polarize as strongly as those in active infection (FDR = 0.008) (Fig. 5—top panel). Though dormant infection M1 polarization was more similar to active infection than uninfected samples, it is possible that this is reflective of a less effective M1 macrophage response in the context of a dormant infection. Of note, degree of M2 polarization was not significantly different either macrophage subtype or myeloid dendritic cells between any groups (Fig. 5—bottom panel).

### The periarticular RNA expression during dormant infection is unique

**Fig. 6:**
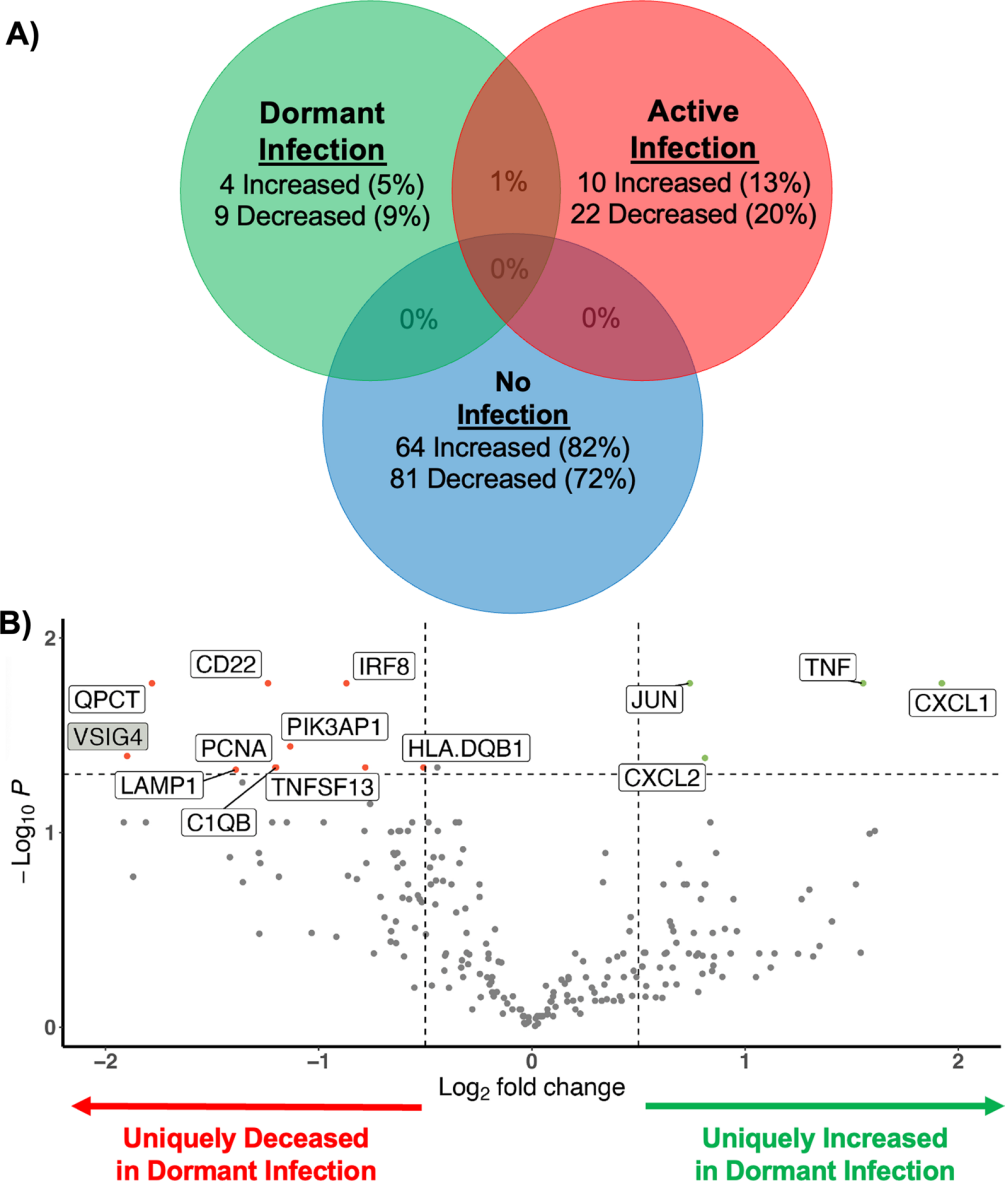
**A** The Venn diagram looks at unique and overlapping gene expressions after bulk transcriptomic analysis, comparing dormant infection (green) to active (red) and no infection (blue). **B** Volcano plot highlighting the transcriptomic changes of the 13 unique (white boxes) and 1 overlapping (grey box) genes expressed during dormant infection (red dot with transcript labeled; the green and grey dots represent transcripts that did not reach significance) when compared to samples with and without an infection

Bulk periarticular RNA transcriptomic analysis further supported these findings, revealing significant differences in gene expression between dormant infections and uninfected joint replacements. This analysis identified one gene (VSIG4) shared between dormant and active infection at the tissue level as well as unique set of 13 genes (4 increased, 9 decreased) that distinguish dormant infection from both active and no infection at the tissue level (Fig. 6A). Of particular interest, the expression of 6 genes (VSIG4, LAMP1, QPCT, C1QB, TNFSF13, and JUN) can be used to distinguish the transcriptomic signature of a dormant infection from an uninfected joint replacement (Fig. 6B) highlighting the potential for these markers in diagnosing a dormant infection.

### TNF and IL17 activation affect neutrophil function during dormant infection

**Fig. 7:**
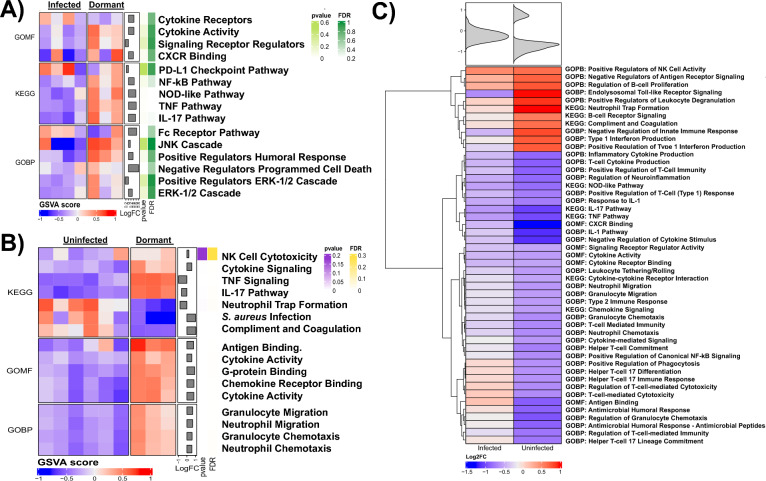
Heatmap of gene set variation analysis (GSVA) using gene ontology (GO) terms and Kyoto Encyclopedia of Genes and Genomes (KEGG) pathways comparing dormant infection to samples **A** with and **B** without an infection based on log_2_ fold change (FC) as well as false discovery rate (FDR). C) Hieratical clustering of GSVA demonstrating comparing dormant infection to active infection (left panel) supports their similarity yielding a normally distributed inflammatory response to infection. However, comparing dormant infection to uninfected controls (right panel) suggest the emergence of a distinct immune signature during dormant infection

Dormant infections showed an innate immune response indicative of defense against bacteria with simultaneous inactivation of the cellular functions required for bacterial eradication. Dormant infections displayed an upregulation of inflammatory pathways involved in the activation of TNF and IL-17 signaling (FDR < 0.01) when compared to uninfected (FDR < 0.01 for both, Fig. 7A) and infected (FDR < 0.01 or both, Fig. 7B) joint replacements. Dormant infections also lead to an upregulation of 4 pathways involved in neutrophil recruitment (granulocyte and neutrophil migration and chemotaxis, FDR < 0.01 for all, Fig. 7B), yet we also saw inactivation of neutrophil function related to extracellular trap formation (FDR < 0.01) and removal or Staphylococcal pathogens (FDR < 0.01, Fig. 7B). This is reinforced by the observation that dormant infections appeared statistically similar to infected joint replacements yet fail to recruit functional neutrophils (Fig. 7C).

### An increase in synovial CXCL5 is associated with dormant infection

**Fig. 8:**
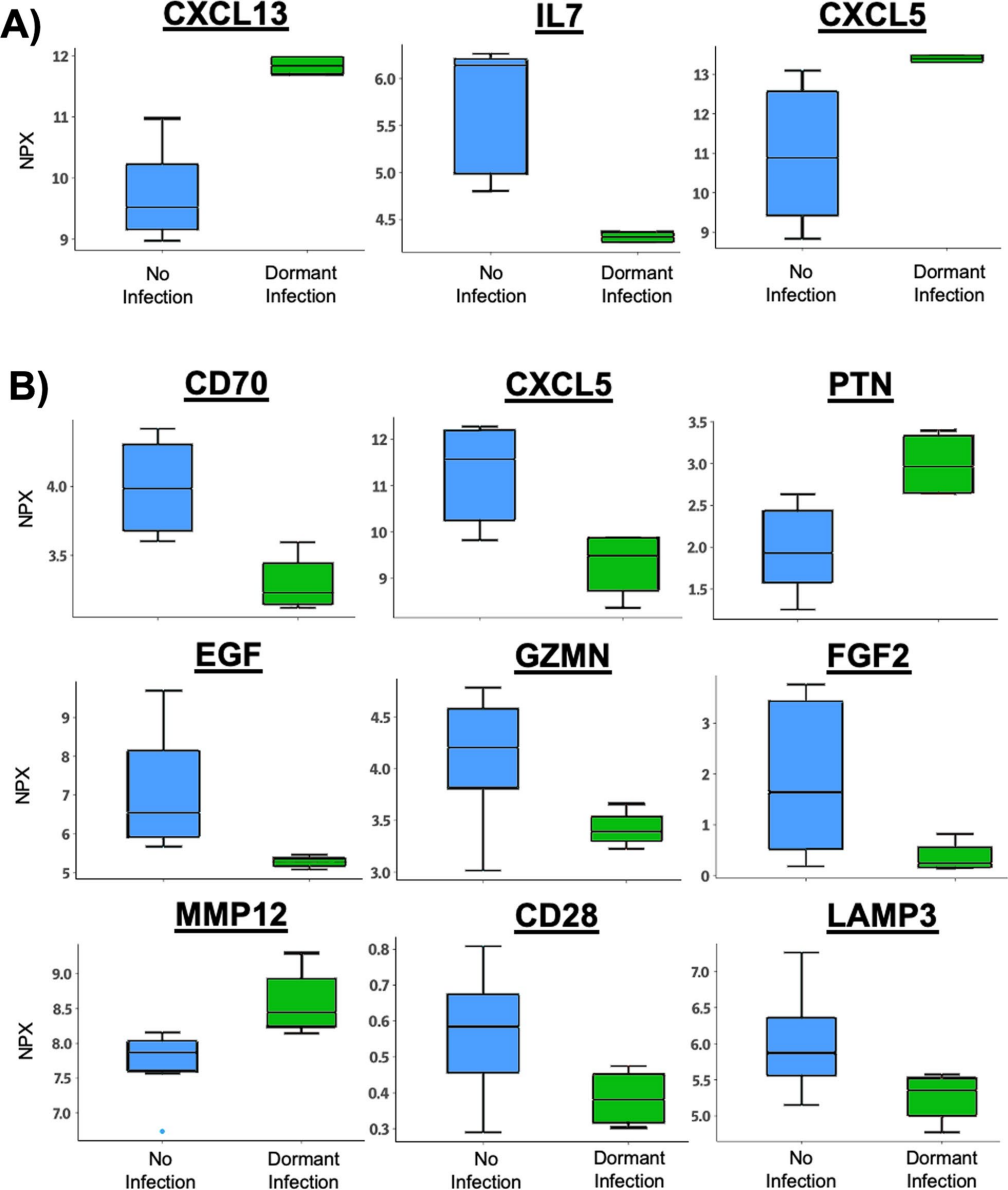
Statistically significant (Welch two-tailed t-test) proteomic changes displayed as median and interquartile range of normalized protein expression (NPX, in log_2_ units) as measured by proximity extension assay on in A) synovial fluid and B) plasma comparing dormant infection (green) to uninfected samples (blue)

Synovial inflammatory proteomics comparing dormant infections and uninfected controls revealed 3 proteins (Fig. 8A). The combination of increased CXCL13 (p = 0.003, t-test) and CXCL5 (p = 0.011, t-test) along with depleted IL7 (p = 0.039, t-test) within synovial fluid strongly. This suggests the establishment of a chronic inflammatory state by the MPS during a dormant infection. The local expression of these 3 proteins within the synovial fluid can be used to distinguish the synovial proteomic signature of a dormant infection from an uninfected joint replacement (Fig. 8A).

### Dormant infections display signs of systemic bacterial tolerance and chronic inflammation

Plasma inflammatory proteomics comparing dormant infections and uninfected joint replacements revealed 9 proteins (Fig. 8B) suggesting a chronic inflammatory state (EGF, GZMN, FGF2, PTN, MMP12) during dormant infection that involves a reduction in neutrophil recruitment (CXCL5, p = 0.006, t-test), antigen presentation (LAMP3, p = 0.047, t-test), and T-cell function (CD28, p = 0.045, t-test; CD70, p = 0.002, t-test) seen during the development of bacterial tolerance. The systemic expression of these 9 proteins within circulating plasma can be used to distinguish the systemic proteomic signature of a dormant infection from an uninfected joint replacement (Fig. 8B).

## Discussion

Our findings underscore the complex immune dynamics in joint replacements, challenging the traditional diagnostic focus on neutrophil responses and pointing towards the importance of a more nuanced understanding of the MPS response after surgery. Definitive diagnostic criteria for infected joint replacements have been difficult to establish. The most widely accepted system is the 2018 update to the MSIS criteria, which has a sensitivity of 97.7% and specificity of 99.5% utilizing a combination of clinical features, serum and synovial analyses, as well as culture results [1]. While these criteria have agreement and even consensus [2], they have failed to account for the possibility of a biofilm-mediated dormant infection. We used paired transcriptomic and proteomic data to establish three novel sets of candidate biomarkers at the tissue, synovial fluid, and systemic level for bacterial tolerance indicative of the immune response to a dormant infection after surgery.

Dormant infections display a local and systemic state of chronic inflammation. Dormant infections display M1 polarization as well as a local increase in CXCL13 and CXCL5, along with depleted IL7 within synovial fluid, strongly suggesting chronic inflammation (Fig. 8A). CXCL13, also known as B lymphocyte chemoattractant (BLC), plays a crucial role in the migration of lymphocytes into inflamed tissues. CXCL5, also called epithelial-derived neutrophil-activating peptide 78 (ENA78), participates in the recruitment of neutrophils. The presence of IL7 depletion indicates a dysregulation of lymphocyte homeostasis, which is common in autoimmune diseases like rheumatoid arthritis. IL7 is also important for the survival and proliferation of T-cells, and its depletion can contribute to the dysregulation of the immune response. These local changes are also coupled with systemic depletion of EGF, GZMN, FGF2, and accumulation of PTN and MMP12, indicating the presence of unresolved tissue damage and repair cycle seen during chronic inflammation. The expression of FN1 by macrophages in uninfected samples suggests the coordination of fibroblast activity involved in tissue healing that is not present during dormant or active infection [82]. The expression of CD63 and S100A10 by myeloid dendritic cells in uninfected samples suggests resolution of toll-like receptor signaling and a reduction inflammatory cell recruitment [83, 84]. HLA and SPP1 expression suggests M2 polarization and antigen presentation may be shifting despite a lock of a change in overall M2 polarization during dormant infection [85, 86].

Dormant infections likely establish bacterial tolerance. We identified strong transcriptomic signs of MPS-mediated bacterial tolerance that extended beyond clinical neutrophil recruitment to neutrophil function, macrophage and dendritic cell differentiation, antigen presentation, and even lymphocyte function [18]. Local macrophage and myeloid expression revealed a process modulating neutrophil recruitment (C1QA, C1QB, LY86, SELL, CXCL5, CCL20, CD14, ITGAM), macrophage polarization (FOSB, JUN), immune checkpoint regulation (IFITM2, IFITM3, CST7, THBS1), and T-cell response (VISIG4, CD28, FYN, LAT2, FCGR3A, CD52) during dormant infection. The systemic depletion of LAMP3 (Lysosomal-associated membrane protein 3) suggests impaired antigen presentation, affecting the adaptive immune response, while the systemic depletion of CD28 and CD70 indicates a decrease in lymphocyte activation and function. Surprisingly, MPS-mediated bacterial tolerance may allow as many as 50% of joint replacements with a dormant infection to be misclassified as “infection-free” prior to re-implantation, thus limiting the prognostic capabilities of all current diagnostic tests that remain over-reliant on the neutrophil response to diagnose late infection in particular [6].

We observed an increase in IL17 activity associated genes in the cohort of patients with a dormant infection, highlighting this cytokine's critical role in both immune defense and pathology. IL17 facilitates the recruitment of neutrophils and other immune cells to infection sites, enhances antimicrobial peptide production, and maintains epithelial barrier integrity, thus playing a pivotal role in controlling infections [87]. However, in chronic infection, persistent IL17 activation can lead to sustained inflammation and tissue damage, contributing to diseases like chronic obstructive pulmonary disease [88] and inflammatory bowel disease [89–93]. IL17 often acts synergistically with TNFα, IL1β, and IL6, amplifying its inflammatory response, while its production is regulated by the IL23/IL17 axis [94, 95]. In the context of dormant infection, we observed increased KEGG pathways representing IL17 and TNFα signaling (Fig. 7C). GOBP pathway comparisons between dormant and uninfected samples suggest a strong upregulation of the granulocyte/neutrophil axis including increased migration and chemotaxis (Fig. 7B). IL17, CXCL1, and CXCL5, which were elevated in dormant infections, are known signals for neutrophil attraction, yet we observe a reduction in signaling related to neutrophil trap formation during a dormant infection when evaluating KEGG pathways (Fig. 7C). This is intriguing since these patients were deemed “infection-free,” yet these patients show a slight elevation in the local and systemic neutrophil response and culture-negative results, indicating low-grade inflammation that does not manifest into full-blown criteria-based infection. Paradoxically, dormant infections also exhibited downregulation of signaling related to neutrophil extracellular trap (NET) formation indicating bacterial regulation of immune-mediated clearance mechanisms (Fig. 7B) [96–102].

**Fig. 9:**
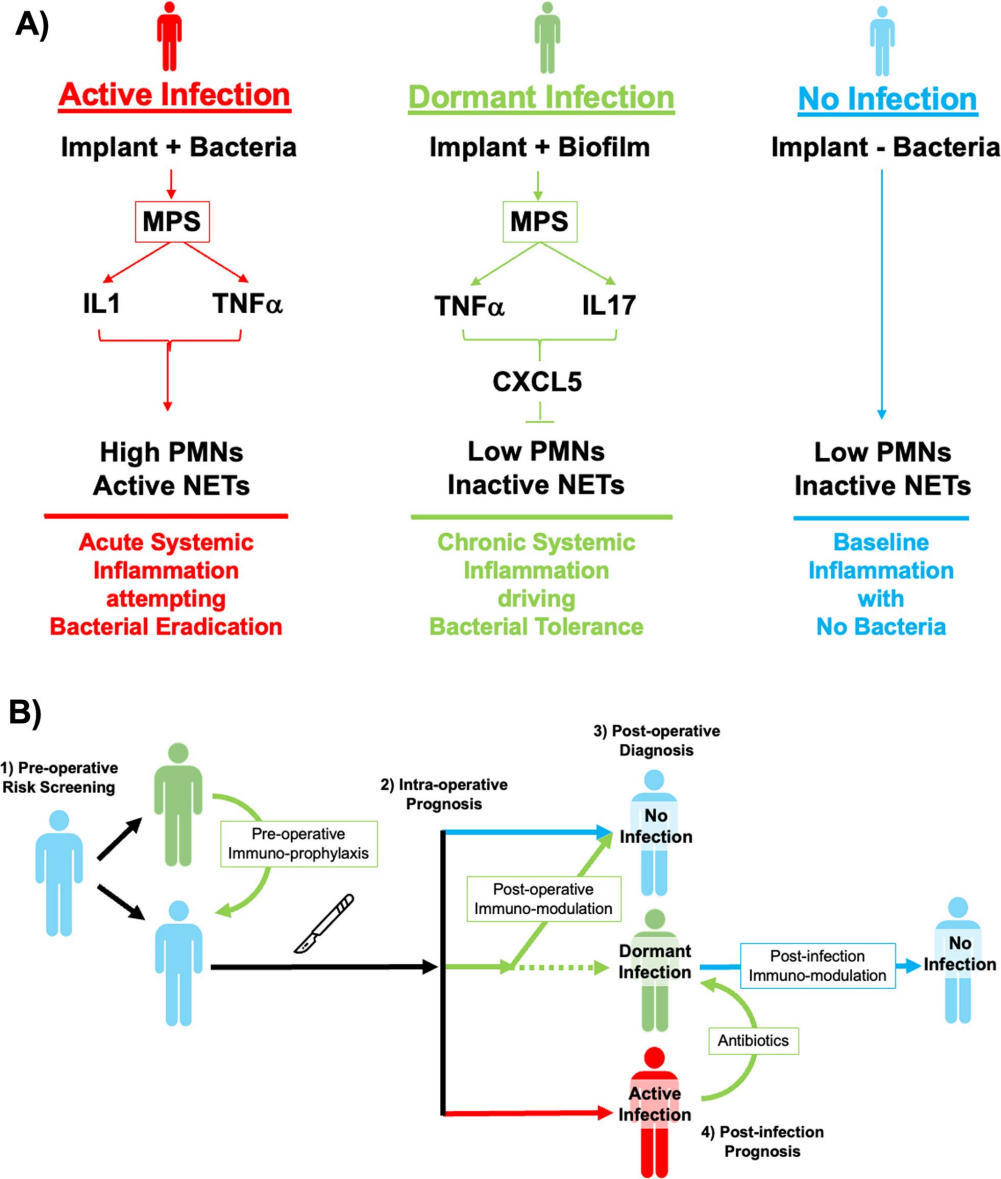
**A** Schematic understanding of dormant infection (green) as an MPS-mediated IL17 response to biofilm inhibiting PMN recruitment and function when compared to patients with (red) or without (blue) and active infection. **B** Schematic understanding of how focusing on the MPS-mediated response as a biomarker of dormant infection (green) enables the screening, diagnosis, and prognosis of dormant infection and identifies a novel paradigm of immuno-prophylactic and immuno-modulation

The release of NETs is an attempt by neutrophils to trap and kill bacterial, viral, parasitic, and fungal pathogens and constrain an emerging infection. NETs also induce uncontrolled inflammation, thrombosis, and tissue damage [103]. NETs are extracellular DNA fibers bearing histones, granular proteins (myeloperoxidase, elastase, and defensins), and cytosolic proteins (calprotectin and cathelicidins) [97, 100, 104]. There is dynamic interplay between the coagulation cascade and NETs [103, 105]. This is of particular interest, because *S. aureus* is proficient in overcoming the innate immune system via is its ability to manipulate the coagulation cascade [106]. *S. aureus* induces fibrin formation by activating prothrombin via the coagulases, von Willebrand factor binding protein and staphylocoagulase [107]. In addition, *S. aureus* binds, activates, and aggregates platelets via its interaction with components of the coagulation system [106, 107]. While NETs trap and kill pathogens, they can also act as a scaffold for biofilm formation [97–99]. It has been hypothesized that neutrophils that are primed to form NETs are no longer able to phagocytize bacteria allowing them to circumvent the innate immune defense [100, 101, 104]. It is also plausible that bacteria promote their own survival by reducing NET formation rather than enhancing it (Fig. 9A). In fact, extracellular adherence protein (EAP) secreted by *S. aureus* can reduce NET formation, destroy released NETs promoting biofilm formation, and interact with NET microbicidal components to limit the antimicrobial potential of NETs [108–113]. Finally, *S. aureus* secretes nucleases (Nuc and Nuc2) that digest extracellular DNA allowing *S. aureus* to evade macrophage immunosurveillance [99, 114–117].

This study has limitations that should be considered when interpreting the results. First, the presence of biofilms in joint replacements directly impacts the detection and analysis of infections since biofilms shield bacteria from immune responses and antimicrobial treatments, potentially leading to an underestimation of infection severity or prevalence in our study [30, 67, 68]. Second, this is a pilot study, and the corresponding sample size is relatively small, with only thirteen patients. This limited sample size can introduce sampling bias, affecting the generalizability of the findings. Larger, more diverse cohorts are needed to validate the results and ensure they are representative of the broader population after multivariate control. Third, while molecular methods offer a culture-independent approach to detecting infections, they may not capture all aspects of the microbial landscape, especially slow-growing or difficult-to-culture organisms. This limitation underscores the need for complementary methods to provide a comprehensive understanding of infection dynamics. Fourth, we did not account for variations in preoperative and postoperative prophylactic treatments and other medical interventions, which could modulate the inflammatory response and influence the study outcomes. Standardizing these factors or adjusting for them in the analysis is crucial for more accurate interpretations. Fifth, computational simulations and modeling could enhance the understanding of the complex interactions within the MPS and the inflammatory response. Simulations could also help predict outcomes and optimize treatment strategies, but this study did not incorporate such approaches. Sixth, we identified associations between the transcriptomic and proteomic profiles of the MPS-based on infection status, but these do not establish causality. Future studies should focus on validating our findings with a larger cohort and longitudinal study to assess the prevalence, characteristics, and progression of dormant infections in joint replacements as well as the possibility of temporal resolution of dormant infection. However, the only re-infection to arise from our cohort was identified after a concomitant dormant infection. Multi-center studies involving diverse patient populations will help establish the sensitivity and specificity of the proposed biomarkers with respect to more traditional diagnostic tests. Targeting the IL17 pathway, both in vitro and in vivo, is required to elucidate its mechanistic role in the establishment and maintenance of a dormant infection. Additionally, the impact of immune checkpoint inhibitors like anti-PD-L1 on enhancing immune responses against dormant infections should be investigated in pre-clinical and clinical models. Integrative multi-omics analyses combining transcriptomic, proteomic, metabolomic, and microbiome data will provide comprehensive insights into molecular mechanisms, aiding in the development of more robust, non-invasive diagnostic tools for dormant infection. The implementation of personalized immune profiles in the determination of risk is essential in this population. However, the real value lies in the ongoing refinement of the MSIS criteria to incorporate MPS-based diagnostic strategies to optimize clinical outcomes (Fig. 9A).

The identification of a pre-disease state is often required to reduce the clinical and monetary impact of final disease state [23, 24]. The onset of many complex diseases initially appears abruptly (e.g., cancer, heart failure, cirrhosis, asthma), but there often exists a reversible pre-disease state prior to the final disease state (e.g., carcinoma in situ, hypertension, high liver enzymes, abnormal pulmonary function tests) [23, 24]. For infected joint replacements, we believe dormant infection represents such a pre-disease state defined by the inability to detect the neutrophil response to an active infection that has the potential for an active infection to emerge later (e.g., when antibiotics stop, immunosuppressives start, or with new surgery/trauma) [3–6, 30, 54, 64–66]. Based on our findings, we estimate that as many as half of all joint replacements may be misclassified as uninfected using neutrophil-dominant diagnostic criteria and may actually harbor persistent MPS-dependent bacterial tolerance response to a biofilm-mediated dormant infection that alters neutrophil recruitment and function. Hence, the accurate identification of dormant infections is of critical importance for cost effective pre-operative screening, establishing intra-operative or post-infectious prognosis, and initiating pre-operative, post-operative, or post-infection immuno-modulatory prophylaxis (Fig. 9B) [24]. It should also be noted that infected joint replacements represent a broader category of implant-associated infections, and the characterization of dormant infections during a joint replacement is likely generalizable to non-orthopaedic implants (pacemakers, abdominal mesh, breast implants) as well as other infections where bacteria use non-viable tissue as an “implant” (osteomyelitis, chronic wounds).

## Supplementary Information

Supplementary Material 1. Table 1. Transcriptomic changes in classically activated M1 macrophages comparing dormant infection to uninfected samples.

Supplementary Material 2. Table 2. Transcriptomic changes in alternatively activated M2 macrophages comparing dormant infection to uninfected samples.

Supplementary Material 3. Table 3. Bulk transcriptomic changes from dormant and uninfected samples.

## Data Availability

The data supporting this study are available upon reasonable request; however, to protect patient confidentiality and comply with ethical guidelines, individual-level information has been anonymized and access is restricted in accordance with data protection policies.
