# Supplementary material for: The mononuclear phagocyte system obscures the accurate diagnosis of infected joint replacements
Source: J Transl Med. 2024 Nov 19;22:1041. doi: 10.1186/s12967-024-05866-5 (PMC11575056; doi:10.1186/s12967-024-05866-5)
Supplement: Supplementary file 1 — Supplementary Material 1. Table 1. Transcriptomic changes in classically activated M1 macrophages comparing dormant infection to uninfected samples. [file 12967_2024_5866_MOESM1_ESM.pdf]

Gene	Base Mean	Fold Change	P-value	P adjusted	Differential
VSIG4	55908	-1.81	8.71E-65	1.95E-62	DOWN
FN1	572640	-2.04	1.15E-62	1.29E-60	DOWN
IGLC3	935	6.69	1.99E-38	1.49E-36	UP
ITGAM	30597	-1.44	3.75E-36	2.10E-34	DOWN
DUSP1	72496	-1.21	5.65E-32	2.53E-30	DOWN
CXCL5	16564	3.6	2.60E-30	9.71E-29	UP
C1QB	316093	-1.12	4.79E-22	1.53E-20	DOWN
LIPA	63260	-1.84	2.43E-18	6.80E-17	DOWN
CD9	41719	-1.24	8.23E-18	2.05E-16	DOWN
IFITM3	17489	1.24	5.87E-17	1.19E-15	UP
SLC7A7	6962	-1.35	8.26E-17	1.54E-15	DOWN
IL3RA	10138	-1.84	6.19E-16	1.07E-14	DOWN
IGKC	724	3.36	7.16E-15	1.14E-13	UP
LAMP1	103017	-1.37	3.07E-14	4.58E-13	DOWN
C10orf54	6538	1.06	1.11E-12	1.46E-11	UP
CD52	36913	-1.64	1.59E-12	1.98E-11	DOWN
SLC25A37	1634	1.22	1.99E-12	2.35E-11	UP
CLEC4E	2471	2.58	3.78E-12	4.23E-11	UP
MMP9	9200	3.19	2.22E-11	2.37E-10	UP
QPCT	4219	-2.03	2.39E-11	2.43E-10	DOWN
CXCL8	47869	1.33	2.80E-10	2.09E-09	UP
FOSB	8275	1.19	7.22E-10	5.22E-09	UP
CXCL2	40092	1.15	4.40E-08	2.99E-07	UP
DUSP4	1421	2.23	1.47E-07	9.43E-07	UP
CD48	2256	1.51	5.74E-07	3.47E-06	UP
IL1RN	3280	1.67	7.98E-07	4.58E-06	UP
LAT2	2370	1.27	8.92E-07	4.99E-06	UP
IER3	15107	1.12	1.02E-06	5.53E-06	UP
PIK3IP1	4364	-1.07	1.04E-06	5.53E-06	DOWN
CXCL3	32379	1.18	1.66E-06	8.34E-06	UP
KCNE3	1834	-1.38	1.67E-06	8.34E-06	DOWN
PRDM1	1154	1.62	6.00E-06	2.69E-05	UP
CCR7	800	2.12	2.08E-05	8.61E-05	UP
LY86	2936	1.12	2.58E-05	1.02E-04	UP
ALAS2	1761	4.62	3.90E-05	1.51E-04	UP
IL1B	2452	1.92	7.30E-05	2.59E-04	UP
TLR8	1621	-1.25	9.90E-05	3.41E-04	DOWN
CXCL1	2264	2.22	1.08E-04	3.66E-04	UP

RNASE2	420	1.54	1.10E-04	3.67E-04	UP
DPP4	1621	1.57	2.02E-04	6.37E-04	UP
IL15RA	606	1.59	3.11E-04	9.06E-04	UP
CLEC10A	1360	1.56	3.57E-04	1.01E-03	UP
TBX21	266	2.42	7.55E-04	2.09E-03	UP
CCL4	6028	1.64	9.38E-04	2.56E-03	UP
TNF	657	1.78	1.38E-03	3.73E-03	UP
CD28	408	-2.3	1.59E-03	4.24E-03	DOWN
IL12RB1	390	1.45	1.75E-03	4.61E-03	UP
LAT	609	1.09	1.88E-03	4.84E-03	UP
IL1R2	264	1.33	1.99E-03	5.06E-03	UP
PCNA	2567	-1.79	2.45E-03	6.16E-03	DOWN
S100A12	421	2.69	3.13E-03	7.80E-03	UP
STAT4	299	1.66	3.99E-03	9.82E-03	UP
CCL20	6886	2.25	4.36E-03	1.03E-02	UP
ITGA4	395	1.37	5.71E-03	1.31E-02	UP
KIAA0101	625	1.06	7.19E-03	1.62E-02	UP
CD72	1254	1.04	1.45E-02	3.07E-02	UP
FAS	717	1.29	1.48E-02	3.11E-02	UP
IL7R	4527	1.18	1.94E-02	3.98E-02	UP
UBE2C	583	1.44	1.97E-02	4.02E-02	UP
CD209	457	1.38	2.39E-02	4.74E-02	UP
