# Supplementary material for: The mononuclear phagocyte system obscures the accurate diagnosis of infected joint replacements
Source: J Transl Med. 2024 Nov 19;22:1041. doi: 10.1186/s12967-024-05866-5 (PMC11575056; doi:10.1186/s12967-024-05866-5)
Supplement: Supplementary file 2 — Supplementary Material 2. Table 2. Transcriptomic changes in alternatively activated M2 macrophages comparing dormant infection to uninfected samples. [file 12967_2024_5866_MOESM2_ESM.pdf]

Gene	Baseline	Fold	P-Value	P adjusted	Differential
C1QB	23270	-2.83	1.64E-15	1.91E-13	DOWN
IER3	744	2.25	2.77E-09	1.24E-07	UP
LAMP1	12391	-1.52	3.20E-09	1.24E-07	DOWN
IFITM3	3705	3.04	4.72E-09	1.37E-07	UP
FN1	78622	-2.77	6.91E-09	1.60E-07	DOWN
FYN	805	1.87	2.14E-08	4.15E-07	UP
TNFSF13	1556	-2.18	1.96E-07	3.25E-06	DOWN
JUN	2015	3.58	4.40E-07	6.39E-06	UP
ITGAM	3537	-1.43	3.78E-06	4.87E-05	DOWN
SLC25A37	269	1.89	7.33E-06	8.50E-05	UP
LAT2	332	3.06	1.91E-05	2.02E-04	UP
FCGR3A	7128	-2	2.62E-05	2.54E-04	DOWN
ITGAE	865	-1.35	3.07E-05	2.74E-04	DOWN
FOSB	2138	2.87	1.38E-04	1.07E-03	UP
C10orf54	865	1.01	1.77E-04	1.29E-03	UP
SELL	181	2.57	2.35E-04	1.52E-03	UP
LY86	323	2.32	2.36E-04	1.52E-03	UP
SLC7A7	720	-1.78	2.94E-04	1.80E-03	DOWN
CD52	4911	-1.58	3.83E-04	2.22E-03	DOWN
IFITM2	648	1.47	4.50E-04	2.37E-03	UP
C1QA	10856	-2.13	4.45E-04	2.37E-03	DOWN
CD9	6271	-1.32	4.71E-04	2.38E-03	DOWN
ADA	560	1.55	1.21E-03	5.84E-03	UP
CXCL5	420	2.59	1.50E-03	6.02E-03	UP
VPS28	875	1.04	1.51E-03	6.02E-03	UP
CTSD	13258	-1.15	1.49E-03	6.02E-03	DOWN
CD14	5394	-1.71	1.30E-03	6.02E-03	DOWN
LIPA	8042	-2.03	1.58E-03	6.13E-03	DOWN
VEGFA	1382	1.24	1.71E-03	6.38E-03	UP
DOCK8	274	-1.08	2.06E-03	7.47E-03	DOWN
ITGB2	16687	-1.05	2.66E-03	9.35E-03	DOWN
CXCL2	1299	1.76	9.45E-03	3.10E-02	UP
THBD	799	1.11	9.61E-03	3.10E-02	UP
CXCL8	1361	1.35	1.25E-02	3.72E-02	UP
LILRB4	2283	-1.1	1.31E-02	3.80E-02	DOWN
IRF8	835	-1.22	1.65E-02	4.67E-02	DOWN
