# Supplementary material for: The mononuclear phagocyte system obscures the accurate diagnosis of infected joint replacements
Source: J Transl Med. 2024 Nov 19;22:1041. doi: 10.1186/s12967-024-05866-5 (PMC11575056; doi:10.1186/s12967-024-05866-5)
Supplement: Supplementary file 3 — Supplementary Material 3. Table 3. Bulk transcriptomic changes from dormant and uninfected samples. [file 12967_2024_5866_MOESM3_ESM.pdf]

| Gene | Baseline | Fold | P-value | P adjusted | Differential |
|---|---|---|---|---|---|
| VSIG4 | 76829 | -2.45 | 1.02E-61 | 2.50E-59 | DOWN |
| FN1 | 886896 | -2.54 | 7.70E-58 | 9.47E-56 | DOWN |
| C1QB | 409741 | -1.57 | 6.66E-49 | 5.46E-47 | DOWN |
| IFITM3 | 26543 | 1.39 | 3.76E-37 | 2.31E-35 | UP |
| IL3RA | 14325 | -1.82 | 2.66E-36 | 1.31E-34 | DOWN |
| ITGAM | 42456 | -1.83 | 1.33E-34 | 5.45E-33 | DOWN |
| RNASE2 | 673 | 2.13 | 6.92E-30 | 2.43E-28 | UP |
| DUSP1 | 108643 | -1.11 | 8.94E-29 | 2.75E-27 | DOWN |
| PIK3AP1 | 15327 | -1.49 | 1.80E-26 | 4.92E-25 | DOWN |
| CD2 | 1204 | 4.22 | 1.32E-24 | 3.24E-23 | UP |
| SLC7A7 | 10078 | -1.67 | 4.93E-24 | 1.10E-22 | DOWN |
| CD9 | 64870 | -1.82 | 5.66E-24 | 1.16E-22 | DOWN |
| CST7 | 4029 | 4.75 | 5.37E-22 | 1.02E-20 | UP |
| CXCL5 | 16808 | 3.29 | 7.78E-20 | 1.37E-18 | UP |
| TNFSF13 | 21220 | -1.02 | 3.97E-19 | 6.51E-18 | DOWN |
| LAMP1 | 146617 | -1.84 | 1.07E-18 | 1.55E-17 | DOWN |
| CD6 | 1005 | 2.62 | 1.55E-17 | 2.11E-16 | UP |
| QPCT | 6729 | -2.26 | 3.63E-17 | 4.70E-16 | DOWN |
| CCR7 | 2683 | 2.96 | 1.37E-15 | 1.68E-14 | UP |
| TLR8 | 2270 | -1.94 | 3.69E-15 | 4.12E-14 | DOWN |
| SLC25A37 | 2144 | 1.17 | 4.06E-15 | 4.34E-14 | UP |
| LIPA | 100850 | -2.42 | 8.05E-15 | 8.26E-14 | DOWN |
| CD163 | 160317 | -1.23 | 1.19E-14 | 1.12E-13 | DOWN |
| IGKC | 1945 | 4.48 | 1.66E-14 | 1.51E-13 | UP |
| CD52 | 58436 | -1.96 | 1.93E-13 | 1.58E-12 | DOWN |
| CXCL1 | 2939 | 2.63 | 6.61E-13 | 4.93E-12 | UP |
| LAT2 | 3102 | 1.42 | 8.33E-13 | 6.03E-12 | UP |
| CD48 | 3441 | 1.45 | 8.87E-12 | 6.06E-11 | UP |
| ITGAE | 9395 | -1.04 | 1.86E-10 | 1.24E-09 | DOWN |
| DUSP4 | 2731 | 2.44 | 3.86E-10 | 2.26E-09 | UP |
| PCNA | 4258 | -1.57 | 5.54E-10 | 3.17E-09 | DOWN |
| S100A12 | 1268 | 3.71 | 1.06E-09 | 5.95E-09 | UP |
| FYN | 6025 | 1.22 | 1.36E-09 | 7.45E-09 | UP |
| FCN1 | 10619 | 3.27 | 1.94E-09 | 1.04E-08 | UP |
| CXCR3 | 906 | 3.63 | 3.20E-09 | 1.64E-08 | UP |
| APOE | 1422 | -1.04 | 3.17E-09 | 1.64E-08 | DOWN |
| CXCL8 | 59301 | 1.31 | 4.03E-09 | 2.03E-08 | UP |
| GNLY | 1343 | 4.71 | 5.25E-09 | 2.58E-08 | UP |

| | | | | | |
|---|---|---|---|---|---|
| CD7 | 478 | 3.67 | 5.94E-09 | 2.86E-08 | UP |
| FCGR3A | 132738 | -1.1 | 9.10E-09 | 4.23E-08 | DOWN |
| FOSB | 12255 | 1.09 | 1.01E-08 | 4.62E-08 | UP |
| PIK3IP1 | 7189 | -1.2 | 1.24E-08 | 5.54E-08 | DOWN |
| DUSP2 | 4292 | 1.65 | 1.37E-08 | 6.01E-08 | UP |
| IL32 | 4423 | 1.95 | 1.60E-08 | 6.91E-08 | UP |
| KCNE3 | 2712 | -1.56 | 1.77E-08 | 7.37E-08 | DOWN |
| IRF8 | 10353 | -1 | 2.08E-08 | 8.55E-08 | DOWN |
| ITGA4 | 795 | 1.87 | 4.04E-08 | 1.60E-07 | UP |
| IL1R2 | 1107 | 2.69 | 6.74E-08 | 2.57E-07 | UP |
| CTSW | 270 | 2.88 | 1.64E-07 | 6.03E-07 | UP |
| TREM1 | 14348 | -1.11 | 1.64E-07 | 6.03E-07 | DOWN |
| BIRC3 | 2069 | 1.25 | 1.68E-07 | 6.06E-07 | UP |
| DOCK8 | 2887 | -1.09 | 2.89E-07 | 1.01E-06 | DOWN |
| MMP9 | 12239 | 2.68 | 3.40E-07 | 1.18E-06 | UP |
| CTSD | 191347 | -1.25 | 4.51E-07 | 1.52E-06 | DOWN |
| TRAC | 505 | 3.85 | 9.84E-07 | 3.19E-06 | UP |
| CLEC4E | 3167 | 2.12 | 9.87E-07 | 3.19E-06 | UP |
| IL1B | 4001 | 2.49 | 1.58E-06 | 5.06E-06 | UP |
| IRF4 | 542 | 3.17 | 1.67E-06 | 5.25E-06 | UP |
| LAT | 984 | 1.26 | 2.27E-06 | 7.08E-06 | UP |
| SELL | 1355 | 2.02 | 4.43E-06 | 1.30E-05 | UP |
| APOBEC3G | 1387 | 1.41 | 4.41E-06 | 1.30E-05 | UP |
| FAS | 1145 | 1.69 | 7.28E-06 | 2.08E-05 | UP |
| IER3 | 18281 | 1.11 | 7.24E-06 | 2.08E-05 | UP |
| CCL5 | 3428 | 2.88 | 1.24E-05 | 3.46E-05 | UP |
| IL15RA | 793 | 1.39 | 1.46E-05 | 4.04E-05 | UP |
| CCL20 | 7652 | 2.34 | 2.58E-05 | 6.84E-05 | UP |
| PRDM1 | 1557 | 1.32 | 2.90E-05 | 7.51E-05 | UP |
| CLEC10A | 3833 | 1.84 | 3.42E-05 | 8.77E-05 | UP |
| CCND2 | 394 | 1.83 | 6.02E-05 | 1.51E-04 | UP |
| CD22 | 724 | -1.42 | 1.24E-04 | 2.96E-04 | DOWN |
| TBX21 | 366 | 2.16 | 2.16E-04 | 4.96E-04 | UP |
| PDCD1 | 58 | 2.41 | 3.26E-04 | 7.36E-04 | UP |
| CD72 | 1815 | 1.27 | 4.25E-04 | 9.33E-04 | UP |
| DPP4 | 1901 | 1.1 | 5.52E-04 | 1.19E-03 | UP |
| THBS1 | 7921 | 1.56 | 5.58E-04 | 1.19E-03 | UP |
| IL6 | 2270 | 2.02 | 7.09E-04 | 1.50E-03 | UP |
| IL1RN | 4329 | 1.29 | 8.04E-04 | 1.68E-03 | UP |

| | | | | | |
|---|---|---|---|---|---|
| CD28 | 517 | -1.41 | 9.30E-04 | 1.91E-03 | DOWN |
| GIMAP5 | 493 | 2.14 | 1.00E-03 | 2.02E-03 | UP |
| STAT4 | 778 | 1.9 | 1.20E-03 | 2.36E-03 | UP |
| IL12RB1 | 517 | 1.49 | 1.58E-03 | 3.07E-03 | UP |
| CHI3L1 | 33071 | -1.46 | 1.68E-03 | 3.21E-03 | DOWN |
| LEF1 | 195 | 2.04 | 2.55E-03 | 4.76E-03 | UP |
| TNF | 757 | 1.36 | 3.59E-03 | 6.55E-03 | UP |
| IL7R | 6749 | 1.43 | 4.76E-03 | 8.37E-03 | UP |
| LIF | 755 | 1.08 | 6.76E-03 | 1.17E-02 | UP |
| ADGRE1 | 332 | 1.82 | 1.13E-02 | 1.93E-02 | UP |
| F5 | 319 | 1.81 | 1.18E-02 | 1.99E-02 | UP |
| MITF | 2227 | -1.03 | 1.18E-02 | 1.99E-02 | DOWN |
| FCER1A | 2410 | 1.79 | 1.50E-02 | 2.44E-02 | UP |
| CD34 | 888 | 1.34 | 2.72E-02 | 4.30E-02 | UP |
| CD244 | 270 | 1.19 | 3.11E-02 | 4.81E-02 | UP |
